# Doppler-ultrasound reference values after pediatric liver transplantation: a consecutive cohort study

**DOI:** 10.1007/s00330-023-09522-2

**Published:** 2023-03-17

**Authors:** Martijn V. Verhagen, Ruben H. de Kleine, Henk Groen, Hubert P. J. van der Doef, Thomas C. Kwee, Robbert J. de Haas

**Affiliations:** 1 Department of Radiology, University of Groningen, University Medical Center Groningen, PO Box 30 001, 9700 RB Groningen, The Netherlands; 2 Department of Hepatobiliary Surgery and Liver Transplantation, University of Groningen, University Medical Center Groningen, Groningen, The Netherlands; 3 Department of Epidemiology, University of Groningen, University Medical Center Groningen, Groningen, The Netherlands; 4 Department of Pediatric Gastroenterology, University of Groningen, University Medical Center Groningen, Groningen, The Netherlands

**Keywords:** Liver transplantation, Child, Ultrasonography, Doppler, Follow-up studies, Reference values

## Abstract

**Objectives:**

Doppler ultrasound (DUS) is the main imaging modality to evaluate vascular complications of pediatric liver transplants (LT). The current study aimed to determine reference values and their change over time.

**Methods:**

A consecutive cohort of pediatric patients undergoing an LT were retrospectively included between 2015 and 2020. Timepoints for standardized DUS were intra-operative and postoperative (day 0), days 1–7, months 1 and 3, and years 1 and 2. DUS measurements of the hepatic artery (HA), portal vein (PV), and hepatic vein(s) (HV) were included if there were no complications during 2 years follow-up. Measurements consisted of: peak systolic velocity (PSV) and resistive index (RI) for the HA, PSV for the PV, and venous pulsatility index (VPI) for the HV. Generalized estimating equations were used to analyze change over time.

**Results:**

One hundred twelve pediatric patients with 123 LTs were included (median age 3.3 years, interquartile range 0.7–10.1). Ninety-five HAs, 100 PVs, and 115 HVs without complications were included. Reference values for HA PSV and RI, PV PSV, and HV VPI were obtained for all timepoints (4043 included data points in total) and presented using 5^th^–95^th^ percentiles and threshold values. All reference values changed significantly over time (*p* = 0.032 to *p* < 0.001).

**Conclusions:**

DUS reference values of hepatic vessels in children after LT are presented, reference values change over time with specific vessel-dependent patterns. Timepoint–specific reference values improve the interpretation of DUS values and may help to better weigh their clinical significance.

**Key Points:**

*• Doppler ultrasound reference values of pediatric liver transplantations are not static but change over time. Applying the correct reference values for the specific timepoint may further improve the interpretation of the measurements.*

*• The pattern of change over time of Doppler ultrasound measurements differs between the hepatic vessel and measurement; knowledge of these patterns may help radiologists to better understand normal postoperative hemodynamic changes.*

**Supplementary information:**

The online version contains supplementary material available at 10.1007/s00330-023-09522-2.

## Introduction

Liver transplantation (LT) remains the only curative treatment option for children with end-stage liver disease. Although graft survival has improved over time [1], pediatric LT is still associated with a higher postoperative complication rate compared to adult LT [2]. Reasons for the higher complication rate are smaller anatomy, discrepancy in vessel size, and challenges associated with childhood diseases and split-liver LT techniques [3].

The main cause of short-term graft loss and mortality is vascular complications such as thrombosis and anastomotic stenosis [4]. With the aim to improve outcomes, short and long-term surveillance imaging is performed to ensure early detection and management of vascular complications [1, 5].

Vascular blood flow is assessed by Doppler ultrasound (DUS) velocity measurements (cm/s) [6, 7]. DUS allows bedside imaging for intra-operative and postoperative assessment of vascular complications after pediatric and adult liver transplantations [8]. Detection of vascular patency with DUS has high specificity for clinically occult hepatic artery thrombosis (99.5%) [5]. Detection of other vascular complications such as anastomotic stenosis may be more challenging and requires a detailed analysis of DUS velocity changes [9].

Currently applied DUS threshold values are largely based on research in adults and do not reflect potential change over time [5, 10]. A study in adults showed that hepatic artery (HA) and portal vein (PV) peak systolic flow velocities (PSV) change over time, and this is may have clinical implications [11]. A study in children that only included postoperative day 1 showed that average DUS velocities and arterial resistive indexes were higher than in adults [12]. This suggests that specific pediatric DUS reference values are necessary.

An abnormal DUS during or after LT warrants further action including imaging (e.g. computed tomography [CT]). Therefore, a better understanding of normal variations of DUS measurements after LT may improve DUS interpretation.

The aim of the present study was to determine DUS reference values for the HA, PV, and hepatic vein(s) (HV) in the immediate postoperative phase, and during short-term and long-term follow-up after pediatric LT. In addition, we aimed to investigate whether there are significant changes over time and whether patient and surgery-related variables affect these measurements.

## Material and methods

### Patients

This single-center retrospective study was performed in the national pediatric liver transplant center of the Netherlands. The study was approved by the local research ethics committee (registry number 201700931), and informed consent was waived. A part of the included population has been previously used to investigate a different study question [13].

Consecutive pediatric LTs performed between April 2015 and December 2020 were eligible for inclusion. LTs were excluded if no DUS images were available. Repeat LT during follow-up was included as separate entries. All patients underwent DUS according to our surveillance protocol. The protocol consists of DUS after finalizing all anastomoses in the operation room with the abdomen open (day 0—open), postoperatively in the operation room and at the intensive care unit (day 0—closed), at postoperative days 1–7, at 1 and 3 months, and at 1 year. In addition, most patients underwent DUS at 2 years as well, resulting in a total of 13 timepoints. Patients fasted before all DUS examinations after the first week.

### Data collection

Data collection and reporting of analysis were performed according to the STROBE guidelines [14]. Data was collected retrospectively from our institutional database. Collected demographic data included: age, gender, and primary liver disease. Disease severity variables were included at listing for LT: Pediatric End-stage Liver Disease score (PELD, under 12 years old), Model for End-stage Liver Disease score (MELD, all ages), international normalized ratio (INR), bilirubin level (umol/L), albumin level (g/L), and creatinine level (umol/L). Included surgical variables were donor type (living or deceased), graft type (segment 2/3, segment 2/3/4, or full-size LT), and vascular anastomosis type (end-to-end, end-to-side, interposition graft, or patch). The hepatic vein piggyback anastomosis at our hospital depends on the graft type. For a segment 2/3 LT (left hepatic vein including variants) the anastomosis is made using a longitudinal vena cava slit, for a segment 2/3/4 (2 hepatic veins) the ‘Nagasaki’ technique is used [15]. In the case of full-size LT (3 hepatic veins), the classic adult piggyback implantation is performed [16].

### DUS measurement

All ultrasound examinations were performed on an Aplio 500 ultrasound machine (Canon) by dedicated radiologists and sonographers. Perioperative and first-week DUS were always performed by the same individual to limit interobserver variation. Images were stored and available in our Picture Archiving and Communication System.

**Fig. 1:**
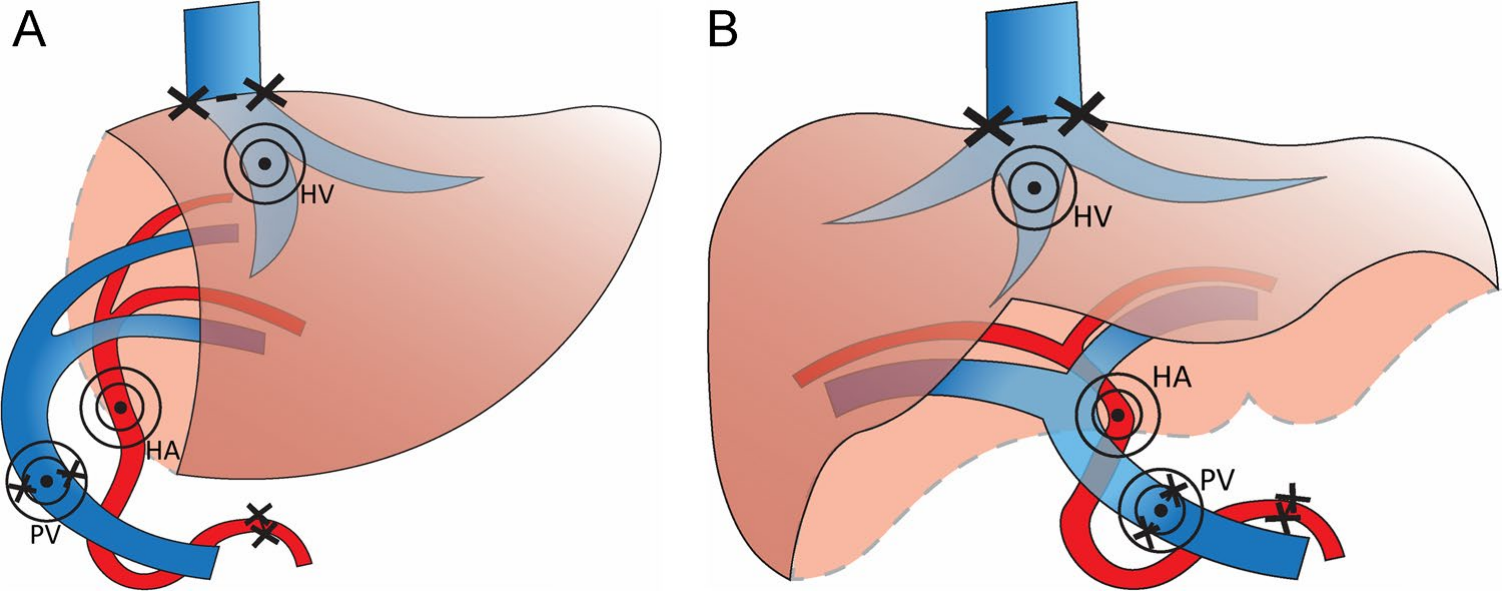
Schematic overview of a segment 2/3 (**A**) and a full size (**B**) liver transplant. The black crosses indicate the vascular anastomoses, the rings indicate the primary sites of Doppler ultrasound sampling. HA, hepatic artery; PV, portal vein; HV, hepatic vein

**Fig. 2:**
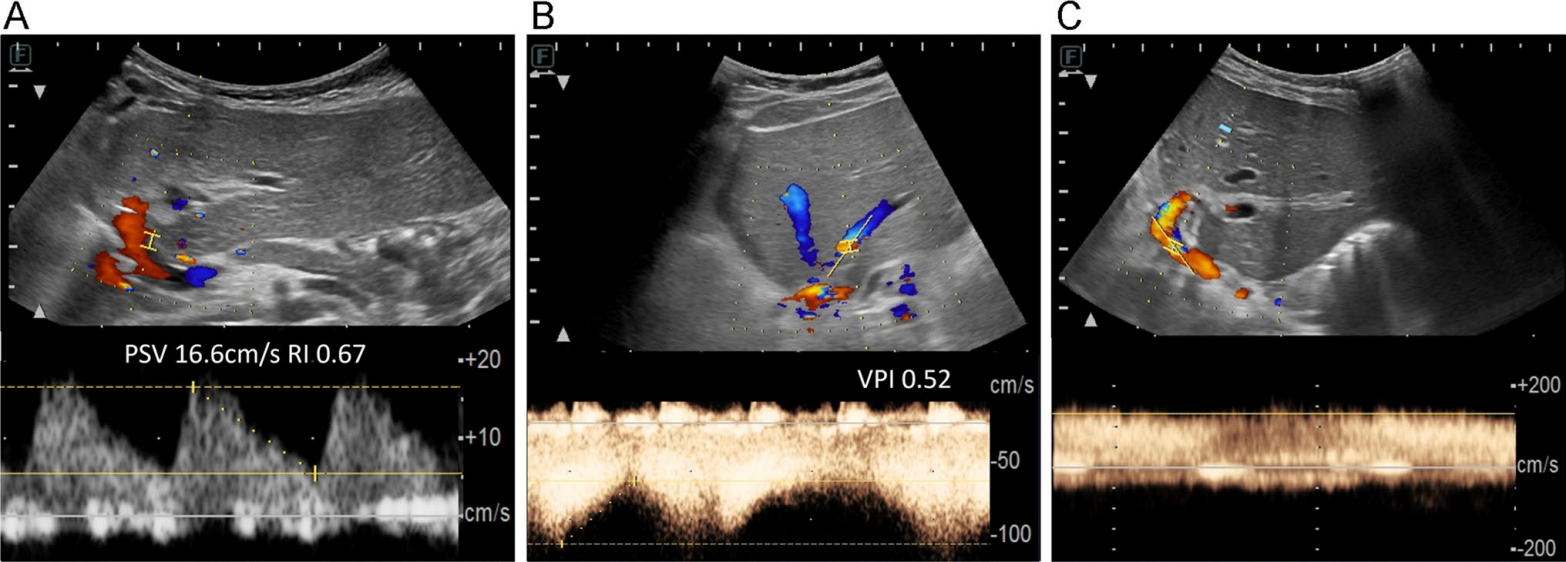
Hilar hepatic artery (**A**) resistive index (RI) and peak systolic velocity (PSV, cm/s), hepatic vein 2 cm from anastomosis (**B**) venous pulsatility index (VPI), and portal vein (**C**) anastomotic PSV

DUS measurements were performed in accordance with published pediatric LT reviews [3, 5] (Figs. 1 and 2):

- HA: Because the HA anastomosis is often not visible, a post-anastomotic HA measurement was performed of the donor artery proximal to the entry into the liver parenchyma. Here, the PSV and RI (PSV minus end diastolic velocity, divided by PSV) were obtained.
- PV: The PSV of the PV was measured at the anastomosis. If the anastomosis could not be clearly defined, the narrowest caliber of the extrahepatic PV was considered the anastomosis. In the case of an interposition graft or patch, the narrowest caliber was sampled.
- HV: The HV waveform was collected from all veins approximately 2 cm proximal to the anastomosis. In the case of multiple hepatic veins, only the best VPI measurement was included (i.e. one measurement per LT per timepoint). VPI was measured as the difference between the maximum (A) and minimum (B) frequency shift divided by (A), according to Chong et al [10]. Although VPI is a continuous parameter and cut-off values for mono-, bi-, and triphasic waveforms are not well established, we considered a VPI of 0 as monophasic, a VPI of > 0 to < 1 biphasic, and a VPI ≥ 1 as triphasic [10].

All DUS images were retrospectively assessed to determine if they fulfilled the above-mentioned criteria. In addition, for all measurements, it was confirmed that the Doppler angle matched the direction of the vessel and that the Doppler measurement was performed with a Doppler angle < 60°, as is standard practice [17]. Only measurements fulfilling these criteria were included. Measurements were registered as missing data if they were not available or did not fulfill the criteria. LTs were not excluded in case of missing values.

DUS measurements were only included for the determination of reference values if there had been no (possible) vessel-specific complications at any timepoint during up to 2 years of follow-up. Possible vascular complications were defined as any possible complication suggested at initial LT or repeat surgery, radiological angiography, CT, or magnetic resonance imaging.

Abnormal DUS measurements, warranting surgical exploration or further imaging, in accordance with pediatric LT reviews were [3, 5]: any thrombosis, a hilar or anastomotic HA PSV > 200 cm/s in combination with a post anastomotic HA tardus parvus waveform (RI < 0.5, systolic acceleration time > 80 ms), a pre- to anastomotic velocity ratio of 3–4 in the PV, a PSV > 125 cm/s in the PV anastomosis, or monophasic (VPI = 0) flow in the HV.

### Statistical analysis

Demographic continuous variables were summarized using median and interquartile range (IQR). Reference values at all timepoints and specified per measurement type and vessel were expressed as percentiles (5^th^, 25^th^, 50^th^, 75^th^, and 95^th^). The range between the 5^th^ and 95^th^ percentile accounted for 90% of the healthy population [18], which we considered a safe reference range suitable for clinical practice. Statistical analysis was performed using SPSS for Windows (version 26, IBM). The level of significance was set at *p* < 0.05.

Generalized estimating equations (GEE) with estimated marginal means (including a 95% Wald confidence interval) were used to analyze changes in DUS values over time in LTs without vessel-specific complications. The potential impact of patient- or transplant-related variables on change over time was assessed using additional GEE models with the following covariates: gender, age subgroups (≤ 2 years, > 2–12 years, > 12 years [19]), graft type (segment 2/3, segment 2/3/4, or full size), donor type (living donor LT, deceased donor LT split liver, deceased donor LT full size), cirrhotic or non-cirrhotic primary disease, and biliary atresia or non-biliary atresia primary disease. First, a model that included the DUS measurements for all timepoints and the respective covariate was constructed without interaction. Next, in case of a significant effect of the covariate (*p* < 0.05), a second model was constructed to test the interaction between time and the covariate. A significant interaction effect was considered to indicate a different course over time of the DUS measurement for levels of the covariate. An exchangeable correlation matrix was used for all analyses with time as a categorical variable. Significant differences are illustrated in graphs. Because biliary atresia represents the largest group in pediatric LT, both significant and non-significant results from the GEE analysis are given.

## Results

### Study population

**Fig. 3:**
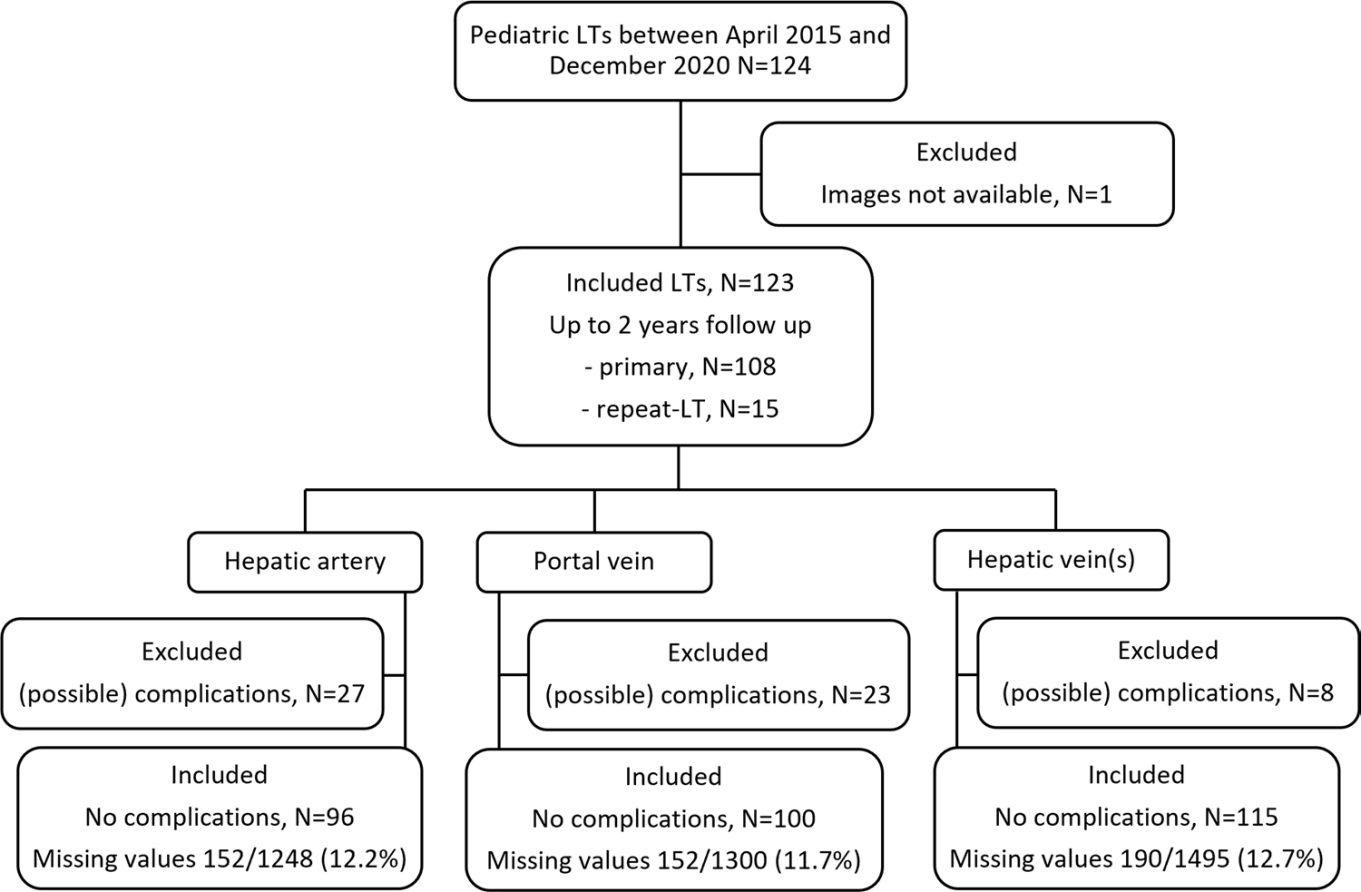
Flowchart study population

**Fig. 4:**
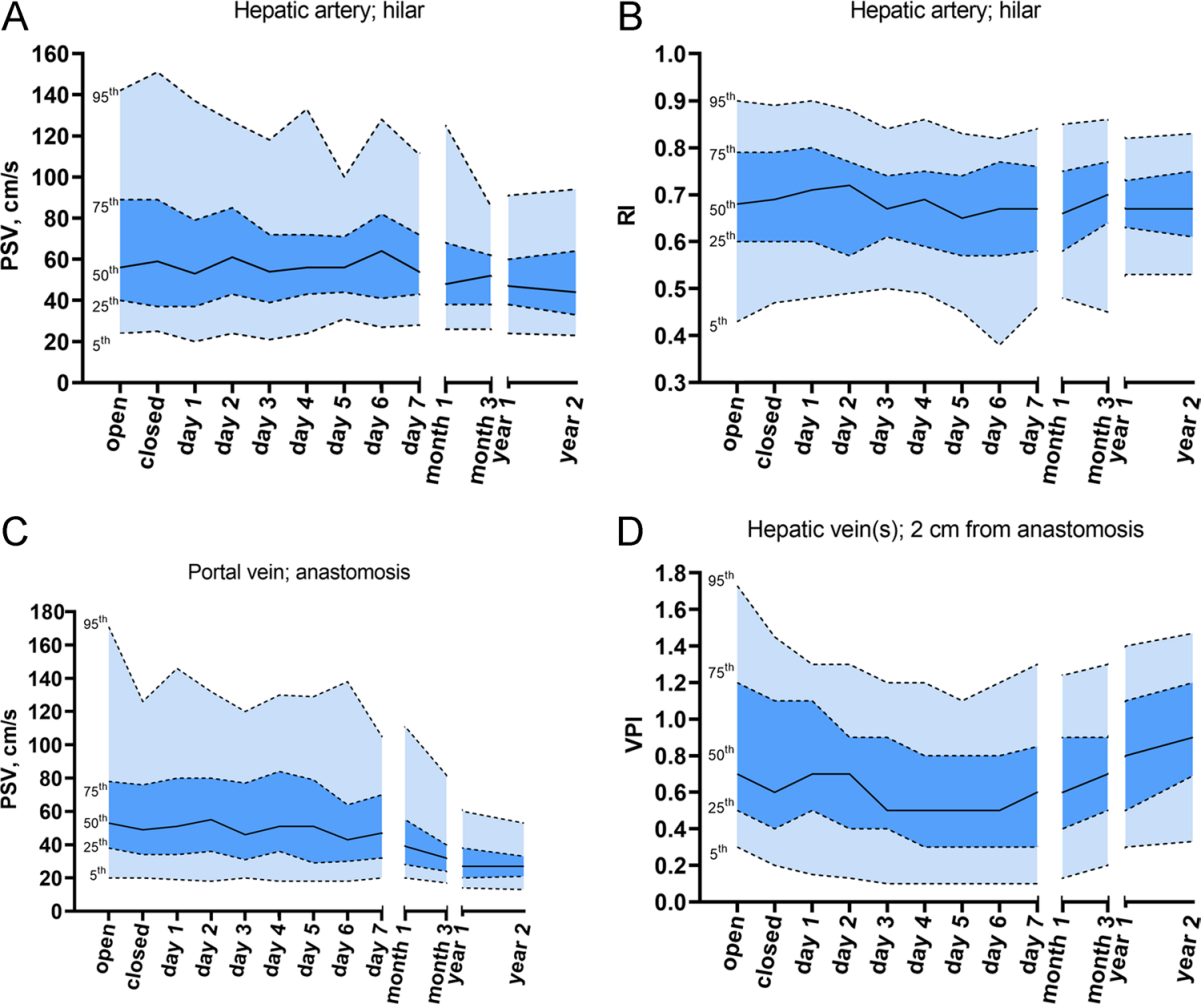
Doppler ultrasound reference values over time, expressed as percentiles, of the hilar hepatic artery **(A** and **B**, peak systolic velocity [PSV]) and resistive index [RI]), portal vein anastomosis (**C**, PSV), and hepatic vein(s) (**D**, venous pulsatility index [VPI]) from day 0 to 2 years after liver transplantation

One hundred and twenty-four consecutive LTs in 112 children were included, of which one patient was excluded because no DUS images were available. The median age at LT was 3.3 years (IQR 0.7–10.1, min–max 0.2–17.8). The mean follow-up was 1.9 years (SD 0.18). Primary liver disease was cirrhosis in 76% (94/123). Eighty-eight percent (108/123) were primary LTs, and 12% (15/123) were re-transplantations. The study flowchart (Fig. 3) illustrates the number of complications per vessel, the subsequent number of included LTs per uncomplicated vessel, and the number of missing cases. Table 1 displays patient characteristics, and supplementary Tables 1 and 2 further display all primary diseases and baseline data. For each DUS variable at each timepoint, the reference values are presented in Table 2 along with the number of included LTs, reference values are illustrated with percentiles in Fig. 4.

**Table 1:** Patient and liver transplantation characteristics

Number of LTs	123 (100%)
Age at LT (years), median (IQR)	3.3 (0.7–10.1)
Gender, male, *N* (%)	68 (55.3%)
Cirrhotic disease, *N* (%)	94 (76.4%)
MELD score*, median (IQR)	17 (12–23.5)
PELD score*, median (IQR)	2.2 (–4.3–11.6)
Bilirubin (umol/L)*, median (IQR)	102 (24.5–265)
Period at waiting list, days, median (IQR)	99 (41–174)
INR*, median (IQR)	1.3 (1.1–1.8)
Albumin (g/L)*, median (IQR)	35 (30–40)
Creatinine (umol/L)*, median (IQR)	21 (14.5–38.5)
Heart beating/non-heart beating donor	
Full size Segment 2 + 3 Segment 2 + 3 + 4	30 (24.4%) 29 (23.6%) 12 (9.8%)
Split liver living donor	
Segment 2 + 3 Segment 2 + 3 + 4	49 (39.8%) 3 (2.4%)

^*^at listing for LT; *INR*, international normalized index; *IQR*, interquartile range; *LT*, liver transplantation; *MELD*, model for end-stage liver disease; *N*, number; *PELD*, pediatric end-stage liver disease

**Table 2:** DUS reference values in children without vessel-specific complications

	Hepatic artery (PSV, cm/s)					Hepatic artery (RI)					Portal vein (PSV, cm/s)					Hepatic vein (VPI)				
	Mean	Median	*N*	IQR	5^th^ – 95^th^	Mean	Median	*N*	IQR	5^th^ – 95^th^	Mean	Median	*N*	IQR	5^th^ – 95^th^	Mean	Median	*N*	IQR	5^th^ – 95^th^
Day 0—open	69	56	78	40–89	24–142	0.68	0.68	79	0.6–0.79	0.43–0.9	64	53	82	38–78	20–171	0.84	0.7	94	0.5–1.2	0.3–1.7
Day 0—closed	70	59	90	37–89	25–151	0.69	0.69	92	0.6–0.79	0.47–0.89	57	49	97	34–76	20–126	0.72	0.6	110	0.4–1.1	0.2–1.5
Day 1	61	53	96	37–79	20–137	0.70	0.71	96	0.6–0.8	0.48–0.9	62	51	99	34–80	19–146	0.75	0.7	114	0.5–1.1	0.2–1.3
Day 2	67	61	94	43–85	24–127	0.69	0.72	94	0.57–0.77	0.49–0.88	62	55	99	36–80	18–132	0.7	0.7	111	0.4–0.9	0.1–1.3
Day 3	59	54	92	39–72	21–118	0.67	0.67	94	0.61–0.74	0.5–0.84	57	46	96	31–77	20–120	0.63	0.5	111	0.4–0.9	0.1–1.2
Day 4	63	56	92	43–72	24–133	0.63	0.69	93	0.59–0.75	0.49–0.86	63	51	97	36–84	18–130	0.56	0.5	108	0.3–0.8	0.1–1.2
Day 5	60	56	84	44–71	31–100	0.65	0.65	89	0.57–0.74	0.45–0.83	57	51	93	29–79	18–129	0.56	0.5	104	0.3–0.8	0.1–1.1
Day 6	65	64	86	41–82	27–128	0.65	0.67	90	0.57–0.77	0.38–0.82	53	43	89	30–64	18–138	0.57	0.5	101	0.3–0.8	0.1–1.2
Day 7	60	54	83	43–72	28–111	0.66	0.67	87	0.58–0.76	0.46–0.84	54	47	91	32–70	20–105	0.6	0.6	105	0.3–0.9	0.1–1.3
Month 1	57	48	83	38–68	26–125	0.67	0.66	83	0.58–0.75	0.48–0.85	48	39	87	28–55	20–111	0.67	0.6	91	0.4–0.9	0.1–1.2
Month 3	53	52	80	38–62	26–86	0.69	0.70	80	0.64–0.77	0.45–0.86	36	32	80	24–40	17–82	0.72	0.7	93	0.5–0.9	0.2–1.3
Year 1	50	47	84	38–60	24–91	0.68	0.67	86	0.63–0.73	0.53–0.82	32	27	85	20–38	14–60	0.81	0.8	98	0.5–1.1	0.3–1.4
Year 2	51	44	54	33–64	23–94	0.68	0.67	55	0.61–0.75	0.53–0.83	29	27	53	21–33	13–53	0.93	0.9	65	0.7–1.2	0.3–1.5

*DUS*, Doppler ultrasound; *IQR*, interquartile range; *N*, number of available measurements at this timepoint; *PSV*, peak systolic velocity; *RI*, resistive index; *SD*, standard deviation; *VPI*, venous pulsatility index

### Hepatic artery hilarPSV (cm/s)

**Fig. 5:**
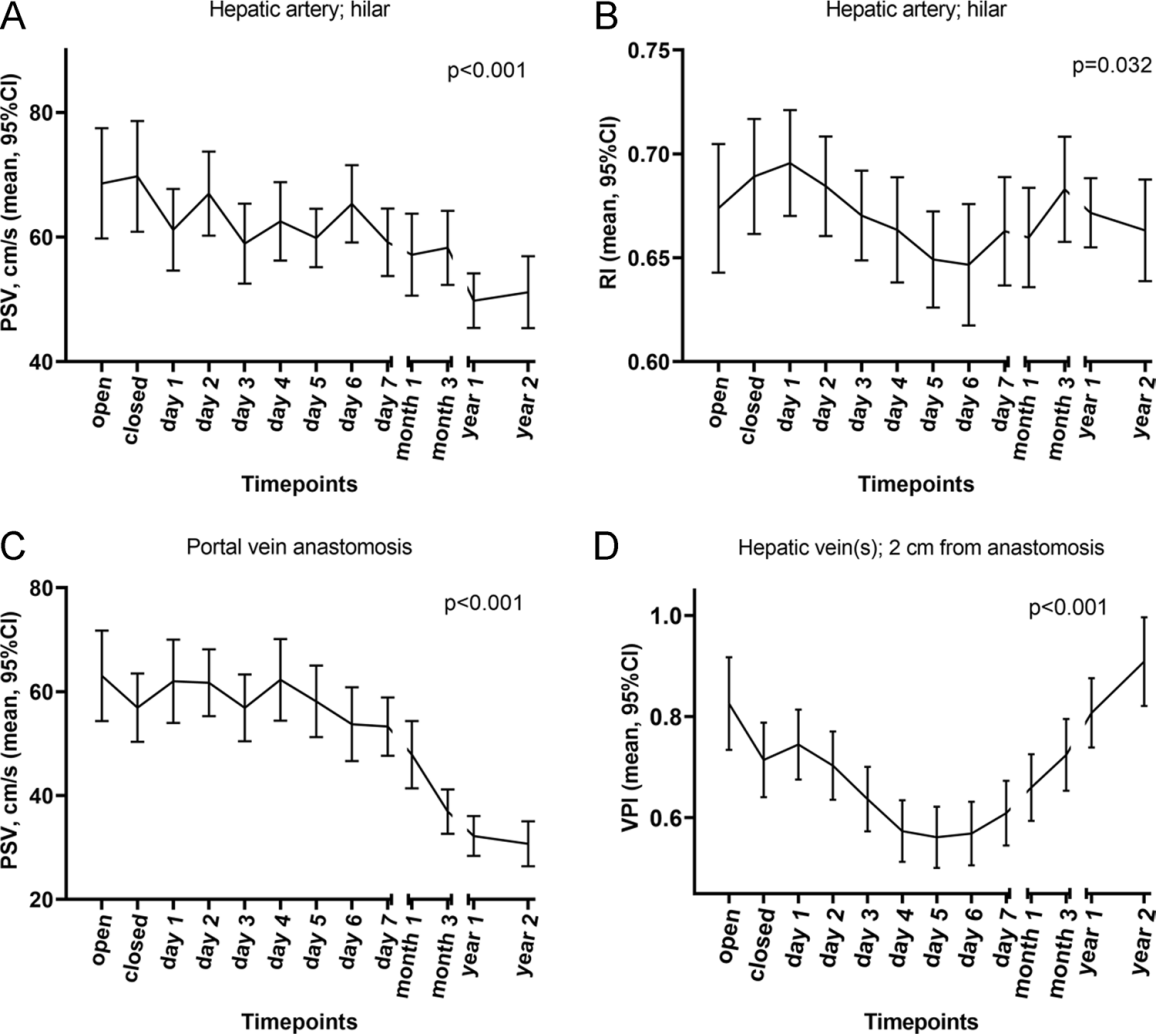
Generalized estimating equations (GEE) with estimated marginal means of change over time, p-values indicate the significance of changes over time. The graphs illustrate the mean and 95% confidence intervals (CI) of the hilar hepatic artery (**A** and **B**, peak systolic velocity [PSV]) and resistive index [RI]), portal vein anastomosis (**C**, PSV), and hepatic vein(s) (**D**, venous pulsatility index [VPI]) from day 0 to 2 years after liver transplantation

HA PSV decreased significantly over time (*p* < 0.001, Fig. 5). Median HA PSV changed from 56 cm/s at day 0 to 44 cm/s at 2 years. The 5^th^–95^th^ percentile interval became smaller over time, from 24 to 142 cm/s at day 0 to 23–94 cm/s at 2 years (Table 2).

**Fig. 6:**
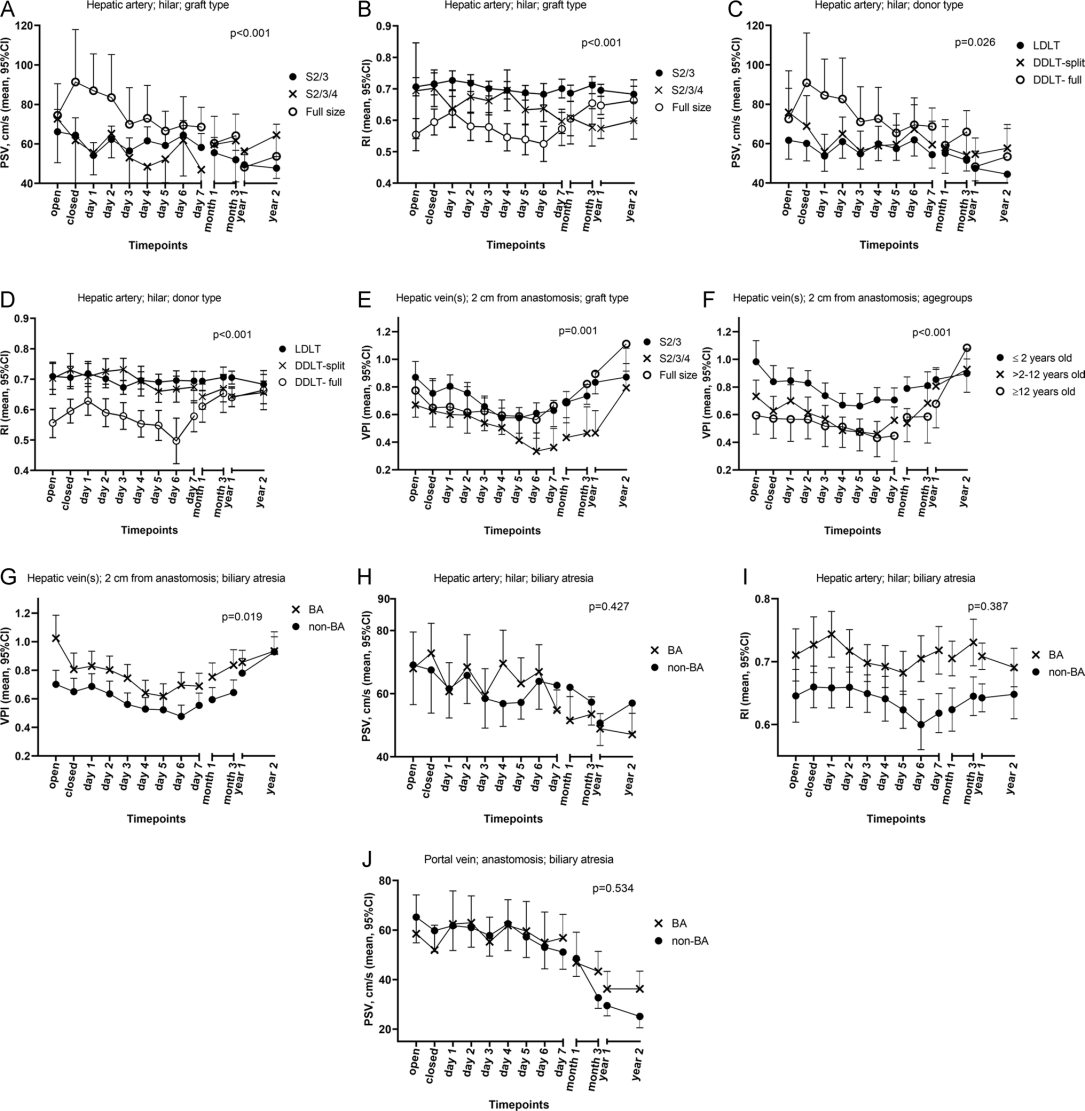
Influence of patient and surgical variables on DUS reference measurements. Generalized estimating equations (GEE) with estimated marginal means. Error bars indicate 95%CI, *p* values indicate the difference in effect of time between groups. DUS measurements of the hilar hepatic artery (**A**–**D**, peak systolic velocity [PSV]) and resistive index [RI]), and hepatic vein(s) (**E**–**G**, venous pulsatility index [VPI]) (**H**–**J**, non-significant GEE analysis of hepatic artery PSV and RI, and portal vein PSV, in patients with biliary atresia (BA))from day 0 until 2 years after liver transplantation. CI, confidence interval; DDLT, deceased donor liver transplant; LDLT, living donor liver transplant

PSV was significantly higher in full-size LTs in the first postoperative days compared to split LTs (*p* < 0.001), but became similar to other graft types at 1 month. A similar significant difference (*p* = 0.026) and pattern were observed when comparing full-size DDLT with split liver DDLT and LDLT (Fig. 6, supplementary table 3).

### Hepatic artery hilar RI

HA RI decreased significantly on postoperative days 5 and 6 (*p* = 0.032, Fig. 5). The change in the median between day 0 and 2 years was negligible (0.68 to 0.67, respectively). The 5^th^–95^th^ percentile interval did become smaller over time, from approximately 0.43–0.90 at day 0 to 0.53–0.82 at year 1 (Table 2).

There was a significantly different pattern of RI change over time between graft types (*p* < 0.001). S2/3 LTs showed almost no change, whereas RIs for S2/3/4 LTs decreased over time, and RIs for full-size LTs were lower than S2/3 and S2/3/4 initially but increased at 3 months (Fig. 6).

RIs of full-size DDLTs were significantly (*p* < 0.001) lower compared to LDLT and split liver DDLTs during the first week, but became similar at 1 month (Fig. 6, supplementary table 3).

### Portal vein PSV (cm/s)

PV PSV decreased significantly over time (*p* < 0.001, Fig. 5), and the median changed from 53 cm/s at day 0 to 27 cm/s at 1 year. The 5^th^ to 95^th^ percentile interval became smaller from 20 to 171 cm/s at day 0 to 14–60 cm/s at 1 year (Table 2). Patient or surgical variables had no impact on PV PSV (supplementary table 3).

### Hepatic vein VPI

VPI changed significantly over time (*p* < 0.001, Fig. 5), and the median changed from 0.7 on day 0 to 0.5 on days 4–6, after which VPI increased to a median of 0.9 at 2 years. The 5^th^–95^th^ percentile interval became slightly smaller over time from 0.3–1.7 at day 0 to 0.3–1.5 at 2 years (Table 2). 

VPI was significantly (*p* < 0.001) higher in children ≤ 2 years compared to > 2 years of age. The difference decreased at 3 months and was similar at 2 years. VPI was also significantly higher in children with biliary atresia (*p* = 0.019), and the difference disappeared at 1 year. Mean VPI was lower in S2/3/4 LTs compared to S2/3 and full-size LTs, being significant (*p* = 0.001) between day 7 and 1 year (Fig. 6, supplementary table 3).

## Discussion

The current study established reference values for all vessels at timepoints up to 2 years after pediatric LT. We showed that DUS values for all vessels change significantly over time, and illustrated that the reference range (5^th^ to 95^th^ percentile) becomes smaller over time, indicating less variation of measurements during follow-up. Patterns of change vary between vessel and measurement types. For VPI, a normal but marked dip at days 4–6 may be seen. Our novel data may help the professionals involved in the care of these children to more accurately weigh the significance of DUS measurements at different timepoints. Our reported upper and lower thresholds for reference values in pediatric LT may lead to changes in practice.

Any DUS value that falls within our suggested reference ranges of the 5^th^–95^th^ percentile is unlikely to be indicative of a clinical problem. However, in case of clinical concerns, a short interval repeat DUS can be considered, especially if the values are near the 5^th^ or 95^th^ percentiles. Values outside the reference range always warrant further attention and the pattern of changes over time should be closely assessed. Short-interval repeat DUS or CT should be considered in these cases.

The upper threshold for a significant HA PSV stenosis as currently applied in practice based on published reviews is PSV > 200 cm/s [3, 20]. However, based on our data, for pediatric LT the 95^th^ percentile for HA PSV of 142–94 cm/s (day 0–2 years, respectively) may already be considered increased and may warrant further clinical discussion, close follow-up, or further imaging. For RI, the generally reported reference values in the literature of 0.50–0.80 were concordant with our data at 1 year post-LT, but we demonstrated a larger reference range between day 0 and 3 months [3].

PV PSV was high in the first postoperative days (95^th^ percentile 171 cm/s at day 0), and when compared to the previously published PV PSV threshold for portal vein stenosis of > 125 cm/s this could result in false-positive interpretations [10]. In contrast, because the upper reference range of PV PSV decreases over time, at 1 year a PV PSV of 60 cm/s instead of 125 cm/s may already warrant further evaluation for stenosis. The observation of a high PV PSV during LT with subsequent decrease over time was similar to previous studies in adults [11, 21]. Various explanations for this initially high and subsequent decrease in PSV have been postulated, including the persistence of increased splanchnic circulation after cirrhosis, loss of sympathetic innervation, and elevated cardiac output [11]. Soft tissue edema and mild kinking may also contribute to early increased velocities. In particular in children, a mismatch between a small native PV (e.g., due to hypoplasia in biliary atresia) and a large adult donor vein, resulting in a surgically more difficult anastomosis, may cause further variability in measurements [22].

Studies involving VPI of the HV after LT are scarce. Instead, the hepatic vein waveforms are generally described using phasicity [3, 12, 23]. However, because VPI describes the HV waveform on a continuous scale, VPI is an objective measurement that can express smaller changes than phasicity, and this is useful for research purposes and clear communication between clinicians. A study by Chong et al in a population of adults and children found that in patients without significant stenosis at venography, the average VPI was 0.75. In addition, a VPI < 0.45 had a sensitivity of 41% and specificity of 95% for stenosis, and they concluded that there was a good correlation between low VPI and a higher chance of stenosis [10]. However, they did not specify the postoperative days on which the measurements were done, which was shown to be important in our study. In addition, we showed that graft type and, in our population, matching type of hepatic vein anastomosis impacts the VPI. By using VPI, we demonstrated a normal dip around postoperative days 4–6. Knowledge of this normal dip, which could also be misinterpreted as pathological deterioration of HV outflow, may prevent unnecessary further imaging or treatment.

Changes in DUS measurements over time are probably multifactorial and may differ per patient. Nevertheless, consideration of these hemodynamic and postsurgical changes remains important when interpreting DUS measurements. Although reference values are useful in clinical practice, individual changes over time may vary depending on specific characteristics, such as age, donor type, and graft type in the current study. To account for this, it may be useful to plot DUS measurements of individual patients over time in order to easily detect deviation from the reference curve, and this may help in deciding if and when to intervene.

The main limitation of our study was the retrospective collection of DUS measurements. Although DUS was performed according to a standardised protocol, by the same investigator in the first week and by a dedicated team afterwards, some interreader variability regarding the exact locations of the DUS measurements in the vessels may have occurred. In addition, the varying investigators after the first week may have resulted in a larger variety of measurements and this may have resulted in a larger 5^th^–95^th^ reference range. Future studies into DUS measurements would benefit from repeat measurements by two investigators, and subsequent determination of interreader variability. However, this may be difficult to ethically justify especially postoperatively because double measurements would prolong the examination of these vulnerable children.

We were also not able to collect sufficient reliable pre- to anastomotic PV ratios, which is an alternative to PV PSV [3]. Because we only investigated the DUS measurements in vessels without possible complications, it remains unclear how much overlap exists between pathological and reference DUS values, and therefore, our data should be viewed with this in mind. Finally, the retrospective methodology resulted in up to 12.7% missing values, spread out over time and over LTs. However, because we included a large LT pediatric population, we believe the results are reliable and highly valuable for clinical practice.

In conclusion, we presented novel DUS reference values for evaluation of blood flow in all hepatic vessels in non-complicated pediatric LT from intraoperative day 0 until 2 years follow-up. Knowledge of timepoint-specific reference values improves the interpretation of DUS values, and thereby, may help clinicians to better weigh their clinical significance. Reference values and their 5^th^-95^th^ range changed over time, and these changes vary per vessel and may depend on the type of graft. We advise using VPI for the evaluation of hepatic venous blood flow to improve structured radiological reporting.

## Supplementary information

Below is the link to the electronic supplementary material.

Supplementary file1 (DOCX 41 KB)

## Abbreviations

DUS: Doppler ultrasound
HA: Hepatic artery
HV: Hepatic vein(s)
LT: Liver transplantation
PSV: Peak systolic velocity
PV: Portal vein
RI: Resistive index
VPI: Venous pulsatility index

## Declarations

### Guarantor

The scientific guarantor of this publication is R.J.de Haas, MD, PhD

### Statistics and biometry

H. Groen, PhD, kindly provided statistical advice for this manuscript.

### Study subjects or cohorts overlap

Some study subjects in the included population have been previously used to investigate a different study question [13].

### Methodology

• retrospective

• cross-sectional study

• performed at one institution
