# Supplementary material for: Doppler-ultrasound reference values after pediatric liver transplantation: a consecutive cohort study
Source: Eur Radiol. 2023 Mar 17;33(9):6404–13. doi: 10.1007/s00330-023-09522-2 (PMC10415481; doi:10.1007/s00330-023-09522-2)
Supplement: Supplementary file 1 — Supplementary file1 (DOCX 41 KB) [file 330_2023_9522_MOESM1_ESM.docx]

| **Supplementary table 1.** Primary diseases and additional baseline study population data. | | | | | | | |
| --- | --- | --- | --- | --- | --- | --- | --- |
|  |  | Age (years) at LT | PELD (<12 y) | MELD (all ages) | Bilirubin level (umol/L) | INR | (possible) vascular complication (any) |
| Primary disease | Number | Mean (SD) | Mean (SD) | Mean (SD) | Mean (SD) | Mean (SD) | N |
| **Cirrhotic liver disease** |  |  |  |  |  |  |  |
| Alagille syndrome | 9 | 7.1(5.1) | 2.4(5.9) | 16.8(4.0) | 197(113) | 1.2(0.1) | 3 |
| Alfa1-antitrypsin deficiency | 5 | 6.4(3.1) | 9.5(13.7) | 21(13.1) | 147(236) | 2.3(1.1) | 4 |
| Auto-immune hepatitis | 2 | 12.9(1.7) | N/A | 15(5.7) | 32(5.7) | 1.8(0.7) | 0 |
| Biliary atresia | 50 | 2.4(3.8) | 3.3(8.1) | 15.9(5.5) | 128(116) | 1.4(0.6) | 20 |
| Cirrhosis secondary to hemophagocytic lymphohistiocytosis | 1 | 4.1 | -0.3 | 14 | 75 | 1.2 | 0 |
| Cirrhosis, unknown cause | 2 | 5.9(3.6) | -5.6(8.6) | 11(5.7) | 98(123) | 1.1(0.1) | 0 |
| Cystic fibrosis | 2 | 17.7(0.1) | N/A | 27.9(8.3) | 230(306) | 1.8(0.3) | 2 |
| Gestational autoimmune liver disease | 1 | 0.3 | 37 | 40 | 444 | 5.2 | 0 |
| Maple syrup urine disease | 2 | 1.8(0) | -17.1 | 6 | 6 | 1 | 2 |
| Nephronophthisis type 3 | 1 | 5.2 | -5.9 | 10 | 35 | 1.1 | 0 |
| Neonatal cholestatic disease of unknown origin | 3 | 3.8(5.2) | 3.7(8.8) | 17(5) | 210(139) | 1.2(0.4) | 2 |
| Neonatal sclerosing cholangitis | 1 | 12.5 | N/A | 11 | 24 | 1.3 | 1 |
| Ornithine deficiency | 2 | 4.3(5.8) | -8.1(1.8) | 8.5(0.7) | 22.5(3.5) | 1.1(0.2) | 1 |
| Primary sclerosing cholangitis | 4 | 13.8(3.1) | 11.7 | 15(5.7) | 131(163) | 1.3(0.1) | 1 |
| Progressive familial intrahepatic cholestasis | 5 | 9.6(6.6) | 0.7(25.8) | 16.5(7.8) | 184(166) | 1.4(0.5) | 4 |
| Sclerosing cholangitis after stem cell transplantation | 1 | 10.9 | -14.8 | 6 | 9 | 1 | 1 |
| Wilson’s disease | 7 | 134(3) | 15(2.6) | 30.8(13.2) | 322(311) | 2.8(1.2) | 2 |
| **Non-cirrhotic liver disease** |  |  |  |  |  |  |  |
| Acute fulminant hepatitis | 14 | 4.7(4.8) | 24.6(14.6) | 30.5(8.8) | 302(225) | 4.0(2.1) | 5 |
| Graft-versus-host-disease | 1 | 10.5 | 9.5 | 20 | 406 | 1.2 | 0 |
| Methylmalonic academia | 2 | 4.5(0.9) | -14.3(1.4) | 18(16.9) | 5.5(4.9) | 1.3(0.4) | 0 |
| Primary hyperoxaluria | 3 | 7.5(2.6) | -7.8(15.8) | 25.5(4.9) | 21.5(24.7) | 1.4(0.6) | 1 |
| Tumor | 5 | 6.6(6.3) | -19.6(1.7) | 16 | 8.2(10) | 1(0.1) | 0 |
| **Total** | 123 | 5.5(5.5) | 4.2(14.3) | 18.9(9.6) | 159(173) | 1.8(1.3) | 49 |
| INR: international normalized ratio; LT: liver transplantation; MELD: model for end-stage liver disease; N: number; N/A: not applicable; PELD: pediatric end-stage liver disease score; SD: standard deviation | | | | | | | |

| **Supplementary table 2.** Types of vascular anastomoses | | | |
| --- | --- | --- | --- |
|  | Full size | Segment 2+3 | Segment 2+3+4 |
| **Hepatic artery** |  |  |  |
| End-to-end, n (%) | 27 (90) | 75 (96.2) | 15 (100) |
| End-to-side, n (%) | 1 (3.3) | 1 (1.3) |  |
| End-to-end with interposition graft, n (%) | 2 (6.7) | 2 (2.6) |  |
| **Portal vein** |  |  |  |
| End-to-end, n (%) | 27 (90) | 55 (70.5) | 14 (93.3) |
| End-to-end with interposition graft, n (%) | 3 (10) | 23 (29.5) | 1 (6.7) |
| **Hepatic veins** |  |  |  |
| Classic piggyback, n (%) | 30 (100) |  |  |
| Nagasaki piggyback, n (%) |  | 78 (100) |  |
| Longitudinal vena cava slit piggyback, n (%) |  |  | 15 (100) |

| **Supplementary table 3.** Results of generalized estimating equations (GEE) models for interaction between patient characteristics and time (complications excluded) | | | | | | | |
| --- | --- | --- | --- | --- | --- | --- | --- |
|  | Hepatic artery | | | Portal vein | | Hepatic veins | |
|  | Number | PSV (cm/s) | RI | Number | PSV (cm/s) | Number | VPI |
| Time (13 levels) | 96 | P<0.001^‡^ | P=0.032^‡^ | 100 | P<0.001^‡^ | 115 | P<0.001^‡^ |
| Gender (male; female) | 56; 40 | P=0.099 | P=0.165 | 57; 43 | P=0.314 | 65; 50 | P=0.652 |
| T*Gender |  | N/A | N/A |  | N/A |  | N/A |
| Age subgroups (≤2; >2-12; ≥12 years old) | 44; 36; 16 | P=0.211 | P<0.001 | 40; 41; 19 | P=0.537 | 52; 45; 18 | P<0.001 |
| T*age subgroups |  | N/A | P=0.137 |  | N/A |  | P<0.001^‡^ |
| Disease type (cirrhotic/non-cirrhotic) | 75; 21 | P=0.589 | P=0.039 | 72; 28 | P=0.021 | 88; 27 | P=0.080 |
| T*disease type (cirrhotic) |  | N/A | P=0.536 |  | P=0.191 |  | N/A |
| Biliary atresia/non-biliary atresia | 42; 54 | P=0.911 | P<0.001 | 36; 64 | P=0.764 | 47; 68 | P=0.001 |
| T*disease type (biliary atresia) |  | (p=0.427) | P=0.387 |  | p=0.534 |  | P=0.019^‡^ |
| Graft type (Full size; S2/3; S2/3/4) | 20; 65; 11 | P=0.016 | P<0.001 | 23; 63; 14 | P=0.052 | 28; 74; 13 | P=0.014 |
| T*graft type |  | P<0.001^‡^ | P<0.001^‡^ |  | N/A |  | P=0.001^‡^ |
| Donor type (LDLT; DDLT-split; DDLT-full size) | 44; 31; 21 | P=0.003 | P<0.001 | 43; 34; 23 | P=0.783 | 49; 37; 29 | P=0.850 |
| T*donor type |  | P=0.026^‡^ | P<0.001^‡^ |  | N/A |  | N/A |
| ^‡^ significant (p<0.05), graphs presented in figures 4 and 5. DDLT: deceased donor liver transplant; LDLT: living donor liver transplant; N/A: not applicable, PSV: peak systolic velocity, VPI: venous pulsatility index, RI: resistive index | | | | | | | |
